# A Review on the Application of Cobalt-Based Nanomaterials in Supercapacitors

**DOI:** 10.3390/nano12224065

**Published:** 2022-11-18

**Authors:** Lin Yang, Qinghan Zhu, Ke Yang, Xinkai Xu, Jingchun Huang, Hongfeng Chen, Haiwang Wang

**Affiliations:** A Key Laboratory of Dielectric and Electrolyte Functional Material Hebei Province, Northeastern University at Qinhuangdao, Qinhuangdao 066004, China

**Keywords:** supercapacitor, cobalt-containing nanomaterials, morphological design

## Abstract

Among many electrode materials, cobalt-based nanomaterials are widely used in supercapacitors because of their high natural abundance, good electrical conductivity, and high specific capacitance. However, there are still some difficulties to overcome, including poor structural stability and low power density. This paper summarizes the research progress of cobalt-based nanomaterials (cobalt oxide, cobalt hydroxide, cobalt-containing ternary metal oxides, etc.) as electrode materials for supercapacitors in recent years and discusses the preparation methods and properties of the materials. Notably, the focus of this paper is on the strategies to improve the electrochemical properties of these materials. We show that the performance of cobalt-based nanomaterials can be improved by designing their morphologies and, among the many morphologies, the mesoporous structure plays a major role. This is because mesoporous structures can mitigate volume changes and improve the performance of pseudo capacitance. This review is dedicated to the study of several cobalt-based nanomaterials in supercapacitors, and we hope that future scholars will make new breakthroughs in morphology design.

## 1. Introduction

### 1.1. Background

Using non-renewable resources such as fossil fuels will cause severe environmental pollution, and their prices are rising yearly due to their dwindling reserves. Therefore, it is urgent to develop sustainable green energy, among which wind and solar energy have been used on a large scale [1]. To better store and transport electricity from sustainable energy sources, energy storage technology has been developed significantly. Rechargeable batteries and supercapacitors (SCs) have been the major chemical energy storage devices.

At present, rechargeable lithium-ion batteries with good safety performance, high voltage and high energy density are widely used. However, with the rising demand for lithium-ion batteries, lithium resources are facing an extremely tight situation. Thus, sodium, an alkali metal, has attracted increasing attention in recent years due to its abundant content and low cost. However, poor cycle performance is still the most significant problem hindering the development of sodium-ion batteries. Compared to rechargeable batteries, SCs have faster charging and discharging processes (SCs: 1–10 s and batteries: 0.5–5 h), higher power density (SCs: 500–10,000 W kg^−1^ and batteries < 1000 W kg^−1^), longer lifetime (SCs > 500,000 h and batteries: 500–1000 h) and safer operation [2,3,4,5]. However, SCs have a disadvantage in terms of low energy density (SCs: 1–10 W h kg^−1^ and batteries: 10–100 W h kg^−1^) [2,6,7,8,9]. To get over the barrier of low energy density, one of the most common approaches is to develop high-performance electrode materials for SCs.

### 1.2. Transition Group Metals Electrode Materials

Transition group metal materials have been widely used as electrode materials for SCs in recent years, and include oxides/hydroxides [10,11,12,13], sulfides [14,15,16,17], phosphides [18], and other categories. Among these materials, RuO_2_, the most representative one, was considered the most desirable pseudocapacitive material for its theoretical specific capacitance (1300–2200 F g^−1^) [19]. However, insufficient resources and the environmental toxicity of RuO_2_ has unfortunately limited its further development [20]. This has led the relevant research on RuO_2_ to its compound materials and other transition group metals to reduce the cost. Among them, cobalt-based materials are promising electrode materials for SCs because of their natural abundance, good cycle stability, abundant electroactive sites, high specific capacitance, and high electronic conductivity. In recent years, various cobalt-based materials, such as Co_3_O_4_, Co(OH)_2_, cobalt-based ternary metal oxides, and sulfides, have been widely studied and many advances have been made.

### 1.3. Contents of This Review

Scholars have done much research on cobalt-based nano-material electrodes. However, their broad application is limited due to low electrochemical potential window, poor structural stability, unsatisfactory cycle stability and low power density. Generally speaking, the morphology, chemical composition and crystal defects of cobalt-based electrode materials have a great influence on the electrochemical performance of energy storage devices. Researchers have explored this issue, including doping other elements, introducing oxygen vacancies, and controlling synthesis conditions to construct different spatial structures of materials to improve the performance of the above electrode materials.

As far as we know, most of the existing reviews classify cobalt-based nanomaterials into a specific class of materials for a brief overview, while few reviews summarize their applications in SC electrodes alone. To promote future breakthroughs in this field, we provide a more comprehensive description of the application of cobalt-based nanomaterials in supercapacitors. Starting from nano-structured cobalt-based materials (cobalt tetroxide, cobalt hydroxide, cobalt-containing ternary metal oxides) and their composites, the application of cobalt-based materials in supercapacitor electrodes is introduced. First, the working principle and classification of SCs are introduced. Second, the applications of cobalt-based nano-compounds in SCs are studied, including the structure and electrochemical properties of cobalt-based nano-materials, the synthesis methods of electrode materials, the construction of different nano-structures and composites with other materials. In addition, the influence of morphology on the properties of cobalt-based nanomaterial electrodes is emphasized. Finally, we look forward to the development and challenges of SCs and cobalt-based materials.

## 2. Cobalt-Based Nanomaterials for SC Applications

With the popularity of mobile electronic devices, electric vehicles, and new energy vehicles, energy storage systems have become an integral part of modern society. Among them, SCs have become electrochemical containers, and have attracted significant attention because of their safe operation, good cycle performance, fast charging capacity and high-power density.

**Figure 1:**
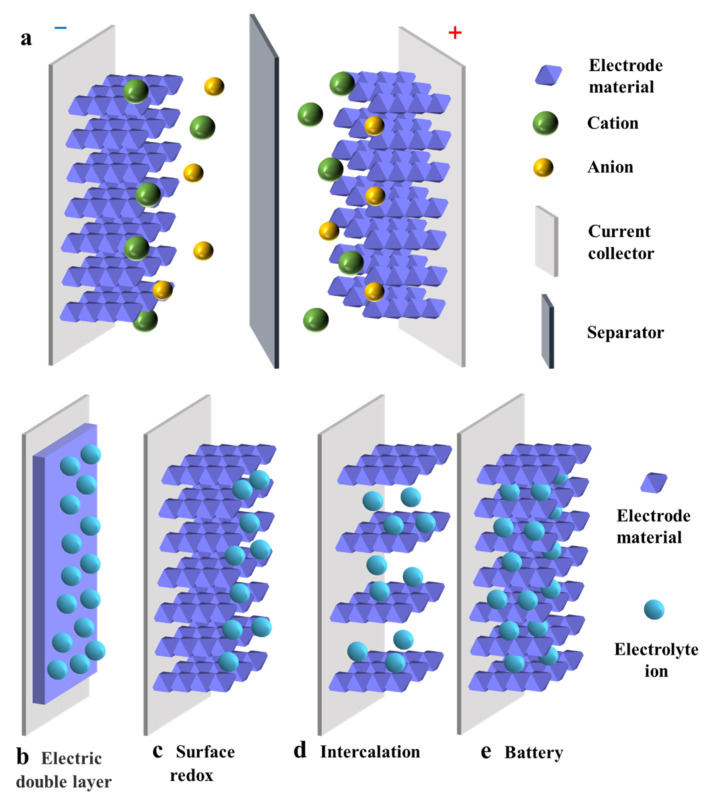
(**a**) Schematic diagram of the structure of an SC and energy storage mechanism of (**b**) an electric double layer capacitor, (**c**) a surface redox capacitor, (**d**) intercalation capacitor and (**e**) a battery-type capacitor.

As shown in Figure 1a, a SC mainly consists of a pair of parallel plate electrodes, an electrolyte solution, electrode materials and an ion-permeable separator [21]. The separator can separate the two electrodes effectively to prevent mutual contact and short circuit [22]. The energy storage mechanism of SCs include (1) reversible ion adsorption and desorption processes between active materials and electrolytes, and (2) reversible faradaic redox reactions during charging and discharging. Furthermore, according to the charge storage mechanism of SCs, they can be divided into three categories: electronic double-layer capacitors (EDLCs), pseudo-capacitors (PCs) and battery-type capacitors. The specific mechanisms of these three types of capacitors are explained below.

EDLC is controlled by reversible adsorption/desorption of electrolyte ions at the electrode/electrolyte interface (Figure 1b), a process involving only the physical adsorption of ions but not any chemical reaction [23]. During the charging process, electrons migrate from the negative electrode to the positive electrode, accumulating positive and negative charges at the two electrodes. Then, the anions in the electrolyte solution move toward the positive electrode and the cations move toward the negative electrode. During the discharge process, the reverse procedure takes place. Since the potential drop is primarily limited to a small range (0.1–10 nm), EDLC has a higher energy density than the conventional capacitor, and its capacitance is related to the interface area of the electrodes. Therefore, common electrode materials mainly include porous carbon-based electrode materials with high specific surface area [24,25,26]. However, due to the absence of Faraday redox reactions in the energy storage process, the charging mechanism confines the capacitance to a lower range, exhibiting a higher power density but lower energy density and specific capacitance.

Based on the Faraday redox reaction, the pseudo-capacitance gives the SCs higher charge storage capacity. Similar to the charging and discharging processes occurring in batteries, the energy storage process in such SCs is a fast reversible Faraday reaction at or near the surface of the active material, but without causing phase changes in the electrode material [22,27]. PCs can be divided into two types: PCs controlled by surface redox reactions (Figure 1c) and PCs controlled by intercalation layers (Figure 1d). For the former PCs, during the redox pseudo-capacitance process, electron transfer occurs when ions in the electrolyte solution are attracted to or near the electrode surface. For the latter PCs, electron transfer occurs when ions are transferred into the gap or interlayer of the electrode and layered electrodes expose a larger area in an electrolyte solution. However, electrode materials are prone to shrinkage and expansion during charging and discharging due to the redox reaction at the electrode, leading to poor cycling performance [28]. Both capacitance and energy density of PCs are much larger than those of EDLCs. This is mainly attributed to the unique charge storage mechanism of the Faraday redox reaction rather than the fully reversible physical charge/discharge processes.

Battery-type SCs (their structures are shown in Figure 1e) are distinguished from PCs by their distinctive feature of exhibiting phase change behavior during charging and discharging [29,30,31]. The charge storage mechanism in battery materials involves the reaction with OH^−^ in alkaline medium, which is controlled by the diffusion of electrolyte ions [31]. Battery-like materials usually have high charge storage capacity. However, the slow phase change of the material during charging and discharging reduces its kinetic performance, making its multiplicative performance low. In contrast, battery-type materials with unique nanostructures have a high specific surface area, creating great active sites for redox reactions and providing a shorter distance for the diffusion of electrolyte ions. Moreover, the rapid phase transition of battery-like materials during charge storage is mitigated by designing their nanostructures.

Transition metal oxides are widely studied as SC electrode materials because they possess higher energy density than carbon materials due to the Faraday electrolysis reaction involved in the electrochemical process. Among them, cobalt nanomaterial is a typical transition metal SC material. In recent years, research on SC electrode materials of Co_3_O_4_, Co(OH)_2_, MnCo_2_O_4_, NiCo_2_O_4_, ZnCo_2_O_4_ and their derivatives have been widely reported.

### 2.1. Cobalt Oxide

In recent years, transition metal oxides have attracted more and more attention as electrode materials with ultra-high electrochemical activity for SCs [32,33,34,35,36,37,38]. Among various transition metal oxides, Co_3_O_4_ electrode materials and related composites have been widely studied because of their high specific capacitance, low price, and environmental friendliness. In addition, the Co_3_O_4_ electrode material, with special microstructure and morphology, has excellent electrochemical capacitance behavior.

At present, several processes are used to prepare Co_3_O_4_, the common ones being hydrothermal [39,40], electrochemical deposition [41], thermal decomposition [42], and sol-gel methods [43]. The hydrothermal method is a process in which the dissolution and recrystallization of insoluble substances occurs in a closed reactor at high temperature and pressure. Experimental parameters, such as temperature, time and molar ratio of additives, have been found to have a significant effect on the morphology of the product [44]. Electrochemical deposition is another important method to prepare electrode materials. During the deposition process, electrical energy can provide a strong driving force for the redox reaction, thus ensuring the uniform growth of electrode materials on conductive substrates, such as stainless steel, nickel foam, and carbon cloth [45,46,47]. Meanwhile, the conductive substrate is used as the working electrode, and deposition conditions such as scan rate, number of cycles, electrolyte concentration and pH are used as control parameters to achieve high surface area and uniform deposition. On the other hand, the thermal decomposition method usually relies on the conversion of certain substances at high temperatures to achieve the modification of electrode materials. This avoids complex multiple synthesis steps and minimizes the use of solvents, making it simple and environmentally friendly. As for the sol-gel method, the process can be described as follows: precursors such as metal alcohol salts or inorganic compounds are hydrolyzed under certain conditions to form a stable and transparent sol system, then are agglomerated into a gel, and finally dried and sintered to form a solid. The advantages of this method are low reaction temperature, easy control of the reaction, and high homogeneity of the sample down to the molecular or atomic level. The shape and size of the nanoparticles are usually controlled by adjusting the ratio of raw materials and the initial pH of the solution.

#### 2.1.1. Co_3_O_4_

As mentioned above, Co_3_O_4_ as a transition metal oxide, has a theoretical specific capacitance of 3560 F g^−1^, good reversibility, and excellent electrochemical properties [48]. Therefore, it is one of the most attractive electrode materials for SCs. However, the capacitive degradation of Co_3_O_4_ at high current densities results in its poor reversibility [49,50]. This phenomenon leads to the actual obtained Co_3_O_4_ specific capacitance being much lower than the theoretical value, so the application of Co_3_O_4_ in SCs is severely limited. It has been reported that the electrochemical performance of Co_3_O_4_ can be greatly improved by regulating the micromorphology of Co_3_O_4_. 

**Figure 2:**
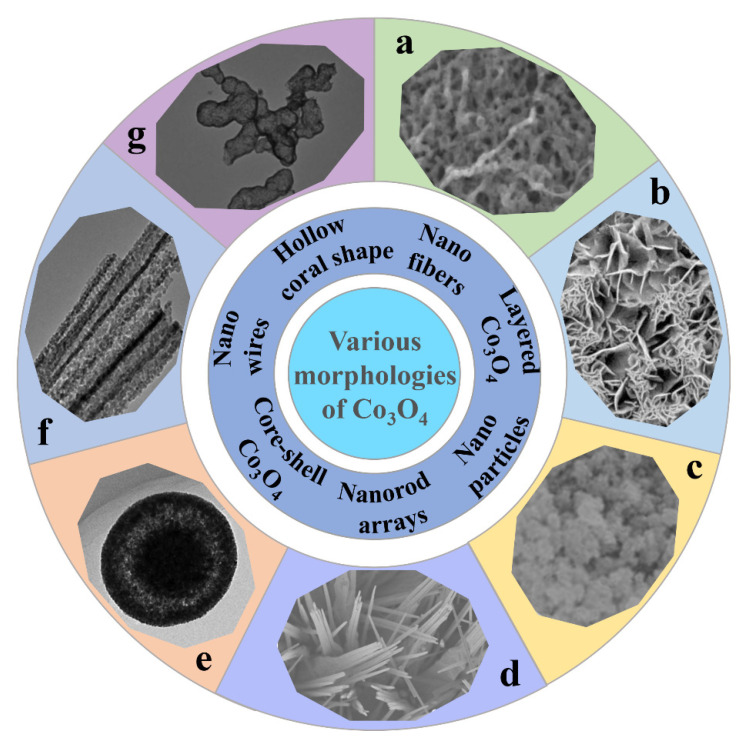
Various morphologies of Co_3_O_4_. (**a**) Nano fibers; reprinted with permission from ref. [51]. (**b**) Layered Co_3_O_4_; reprinted with permission from ref. [52]. (**c**) Nano particles; reprinted with permission from ref. [53]. (**d**) Nanorod arrays; reprinted with permission from ref. [54]. (**e**) Core-shell Co_3_O_4_; reprinted with permission from ref. [55]. (**f**) Nano wires; reprinted with permission from ref. [56]. (**g**) Hollow coral shape; reprinted with permission from ref. [57].

In recent years, various morphologies of Co_3_O_4_ have been synthesized by different methods (shown in Figure 2), such as Co_3_O_4_ nanofibers [51], layered Co_3_O_4_ [52], Co_3_O_4_ nanoparticles [53], Co_3_O_4_ nanorod arrays [54], core-shell Co_3_O_4_ [55], porous Co_3_O_4_ nanowires [56], and hollow coral-shaped Co_3_O_4_ [57]. Several Co_3_O_4_ electrode materials with typical morphologies are briefly described below, including their preparation processes, unique spatial structures, and their principles. For the convenience of readers, the electrical property data of these materials is listed separately in Table 1.

Manish Kumar et al. prepared Co_3_O_4_ nanofibers (shown in Figure 2a) by electrospinning technology [51]. Due to the large specific surface area and unique porous network morphology of this structure, the electrolyte solution can better contact with the electrode material. This is conducive to the transport of ions and electrons at the electrode-electrolyte interface, thus accelerating the redox progress. Duan et al. synthesized layered porous Co_3_O_4_ films by a hydrothermal method [52]. As shown in Figure 2b, the prepared Co_3_O_4_ films display a two-layer structure in which the lower structure consists of an array of Co_3_O_4_ monolayer hollow spheres and the upper structure consists of porous mesh-like Co_3_O_4_ nanosheets. The high porosity and large specific surface area provide a short path for ion/electron transfer, and the close contact between the active material and the electrolyte leads to high electrochemical activity, which enhances the pseudocapacitive performance. In addition, the graded porous structure can also moderate the volume changes caused by redox reactions, thus improving cycling performance. Deng et al. synthesized cobalt oxides (Co_3_O_4_ and Co_3_O_4_/CoO) by burning a mixture of Co(NO_3_)_2_·6H_2_O and citric acid (Figure 2c) [53]. They experimentally confirmed that the morphology of the electrode materials could be influenced by adjusting the citric acid/Co(NO_3_)_2_·6H_2_O molar ratio. Based on this, they produced electrode materials with the best performance. As shown in Figure 2d, unique Co_3_O_4_ nanorod arrays were synthesized through a simple chemical bath deposition and annealing process by Chen et al. [54]. Due to their high specific surface area and novel structure, the specific capacitance of Co_3_O_4_ nanorod arrays is high. It was found that Co_3_O_4_ nanorod arrays have good cycling stability, conductivity, and ion diffusion behavior. Liu et al. prepared Co_3_O_4_ mesoporous nanospheres with a homogeneous core-shell by the solvothermal and rapid calcination methods (Figure 2e) [55]. The accumulation density of sub-nanoparticles and the thickness of Co_3_O_4_ shell layer can be controlled by changing the annealing time. Both the tunable mesoporous and core-shell structures can facilitate the ion and electron transport efficiently while adapting to the volume change of the oxide electrode during cycling. Xu et al. successfully prepared one-dimensional porous Co_3_O_4_ nanowires by thermal decomposition of coordination polymers with nitrilotriacetic acid as a chelating agent using a solvothermal method (Figure 2f) [56]. The porous structure of Co_3_O_4_ nanowires consists of many nanoparticles. The special structure maximizes the exposure of the active material to the alkaline electrolyte, resulting in high specific capacity and good cycling stability. Wang et al. obtained hollow coral-shaped Co_3_O_4_ nanostructures by calcining cobalt oxalate precursors in the air (Figure 2g) [57]. The hollow structure allows it to withstand volume changes during the reaction process and thus exhibits excellent cycling performance.

Starting from improving the contact area between electrode and electrolyte, Lu et al. prepared layered Co_3_O_4_ electrode material by combining 2-methylimidazole cobalt salt and electro-spun nanofibers [58]. Its unique three-dimensional (3D) network and nano porous structure reduced the ion diffusion distance and increased the contact area between electrode and electrolyte, thus improving its electrochemical performance. The synthesized Co_3_O_4_ electrode can provide a high specific capacitance of 970 F g^−1^ at a current density of 1 A g^−1^, an energy density of 54.6 W h kg^−1^ at a power density of 360.6 W kg^−1^, and a capacitance retention rate of 77.5% after 5000 cycles at 6 A g^−1^.

The above study showed that the electrochemical performance of Co_3_O_4_ can be significantly improved by adjusting its morphology. By designing a unique structure, the contact area can be increased, and the close contact between the active material and the electrolyte can lead to high electrochemical activity, which enhances the pseudocapacitive performance. In addition, the graded porous structure can also moderate the volume changes caused by redox reactions, thus improving the cycling performance.

#### 2.1.2. Co_3_O_4_ Composites

To further improve the performance of Co_3_O_4_, and meet the needs of various applications, one of the main means is to prepare Co_3_O_4_ composites by anchoring Co_3_O_4_ on a carbon-based material with high electrical conductivity. Among many carbon-based materials, graphene with large specific area, unique mechanical, and excellent electrochemical properties is considered to be an ideal carrier for loading Co_3_O_4_ nanostructures [59]. Therefore, graphene-based Co_3_O_4_ composites have become a research hotspot in recent years. For example, Tan et al. made self-supporting and non-adhesive Co_3_O_4_ nano sheet arrays/graphene/Ni hybrid foams by in-situ synthesis of graphene and Co_3_O_4_ nanosheets on nickel foam [60]. The SEM image shows that the porous structure supported by the composite remain good. At the same time, the substrate is completely covered by Co_3_O_4_ nanosheets and there is no agglomeration. This self-supporting and adhesive free characteristic avoids the disadvantage of the high resistance of traditional graphene-based Co_3_O_4_ composites due to the contact between hybrid particles, additives, adhesives, and collectors. The cycle performance of Co_3_O_4_ nano sheet/graphene/Ni hybrid electrode has been studied. It was found that after 5000 cycles at a current density of 10 mA cm^−2^, it had 112.2% of the initial capacitance. This indicates that the ability of this unique Co_3_O_4_ nano sheet/graphene/Ni hybrid electrode can meet the requirements of good capacity and long cycle life at high current density.

Younis et al. synthesized Co_3_O_4_ nanosheets by one-step electrochemical deposition on carbon foam followed by annealing [41]. The electrochemical properties of the Co_3_O_4_ nanosheets were improved due to the good electrical conductivity of the composite carbon foam. In addition, a dense mesoporous structure could be observed in the SEM images, which may be one of the main reasons for the improved electrochemical properties. Electrochemical tests showed that the prepared Co_3_O_4_ nanosheets had ideal capacitive properties with a maximum specific capacitance of 106 F g^−1^ in 1 M NaOH solution at a scan rate of 0.5 V s^−1^. In this report, the prepared ultrathin nanosheets were simple in process, low in cost, and suitable for industrial applications, which have high reference value.

Introducing oxygen vacancies into transition metal oxides can change their geometric and electronic structures, improve their intrinsic conductivity and electrochemical activity, and improve their properties [61,62,63,64]. For example, Xiang and others prepared Co_3_O_4_ nano sheet electrode materials with different oxygen vacancy content by different reduction methods [65]. They showed that Co_3_O_4_ electrode with high oxygen vacancy content has better electrochemical performance. At the current density of 2 A g^−1^, the capacity retention percentage can reach 95% after 3000 cycles, while the capacitance retention rate of the original Co_3_O_4_ nanosheet electrode was only 90% under the same conditions. This indicates that the introduction of oxygen vacancy can improve the conductivity, increase the capacitance, and significantly improve the electrochemical performance.

Yang et al. used the one-step laser irradiation method for the first time to synthesize ultrafine Co_3_O_4_ nanoparticles/graphene composites with rich oxygen vacancies by laser-induced reduction and fragmentation [66]. Compared with the traditional method, the one-step laser irradiation method is simple, does not need to add reducing agents and additives, and solves the pollution problem of organic additives. At 10 A g^−1^ current density, the capacitance retention of the composites after 2000 cycles could reach 99.3%, while the capacitance retention of porous Co_3_O_4_ nanorods electrodes was only about 84.7%, indicating that Co_3_O_4_ nanoparticles/graphene composites have excellent cycle stability.

### 2.2. Co(OH)_2_

Similar to transition metal oxides, transition metal hydroxides have excellent pseudocapacitive properties [67]. Among them, Co(OH)_2_ has become one of the promising materials in SCs due to its high theoretical capacitance (3460 F g^−1^) and low cost. With electrode materials, reversible redox reactions take place during charge and discharge. The specific process is that Co(OH)_2_ stores charge by participating in the O-H bond breaking and recombination reaction in the electrolyte. The redox reaction can be expressed as [68]:

Co(OH)_2_ + OH^−^ → CoOOH + H_2_O + e^−^ (1)

The oxidation product CoOOH can further undergo a deprotonation reaction and carry out the second redox reaction [22]:

CoOOH + OH^−^ → CoO_2_ + H_2_O + e^−^ (2)

**Figure 3:**
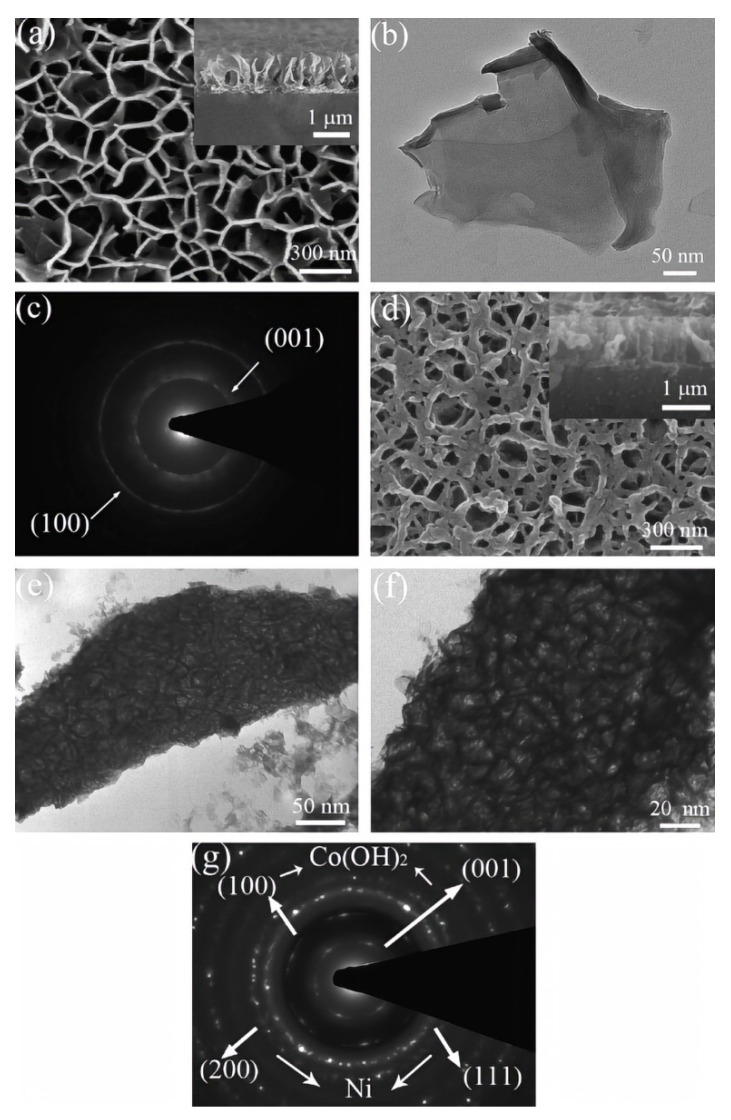
(**a**) SEM image, (**b**) TEM image and (**c**) SAED pattern of a Co(OH)_2_ nanoflake array. (**d**) SEM image, (**e**,**f**) TEM images and (**g**) SAED pattern of a Co(OH)_2_/Ni composite nanoflake array grown on nickel foam. Reproduced with permission from G.X. Pan, Porous Co(OH)_2_/Ni composite nanoflake array for high performance supercapacitors; published by Elsevier, 2012 [69].

Although the theoretical capacitance of Co(OH)_2_ is very high, it is difficult to meet the requirements of fast electron transport rate at high power density because it is a P-type semiconductor. An effective way to alleviate the above problems is to construct conductive matrix hybrid nanostructures of Co(OH)_2_. For example, Pan et al. synthesized Co(OH)_2_/Ni nano-lake array with porous structure by hydrothermal and electrodeposition methods [69]; its microstructure is shown in Figure 3. As a conductive substrate, nickel foam forms a porous conductive network, which can shorten the diffusion path of ions and electrons, and improve the charge efficiency, thus effectively improving the electrochemical performance of SC. When the charge and discharge rate changes from 1 A g^−1^ to 40 A g^−1^, the capacitance retention rate reaches 87.6%, while that of pure Co(OH)_2_ nano-lake array is only 76.4% under the same conditions.

Li et al. prepared a 3D independent Co(OH)_2_/Ni heterostructure electrode by depositing sea urchin-like Co(OH)_2_ microspheres on nickel foam using a one-step hydrothermal method [70]. According to the analysis of its electrochemical performance, the capacitance could reach 1916 F g^−1^ at 10 mA cm^−2^, and 79.3% of the original capacitance was maintained after 5000 charge and discharge cycles at 80 mA cm^−2^ current density. The reason for this decrease in capacitance is that some sea urchin-like Co(OH)_2_ microspheres become inconspicuous rod-like and stacked plate-like CoOOH due to changes in composition and structure during charging and discharging.

To improve the density of SCs while maintaining their flexibility, Zhao and his colleagues deposited Co(OH)_2_ on nickel oxide/hydroxide coated nano porous nickel (np-NiO_x_H_y_@Ni) by electrochemical deposition [71]. Then they successfully synthesized a Co(OH)_2_/np-NiO_x_H_y_@Ni hybrid electrode with a hierarchical porous structure and excellent flexibility. The layered porous structure improves the surface area and effectively promotes ion diffusion. At the same time, the coordination between Co(OH)_2_ and NiO_x_H_y_ electroactive materials significantly improves the electrochemical reaction activity of electrode materials. The capacitance of Co(OH)_2_/np-NiO_x_H_y_@Ni electrode was 1421.1 F cm^−3^ at 0.5 A cm^−3^ current density, and 81.6% of the original capacitance remained after 8000 cycles at 2 A cm^−3^ current density.

### 2.3. Cobalt-Containing Ternary Metal Oxide

**Figure 4:**
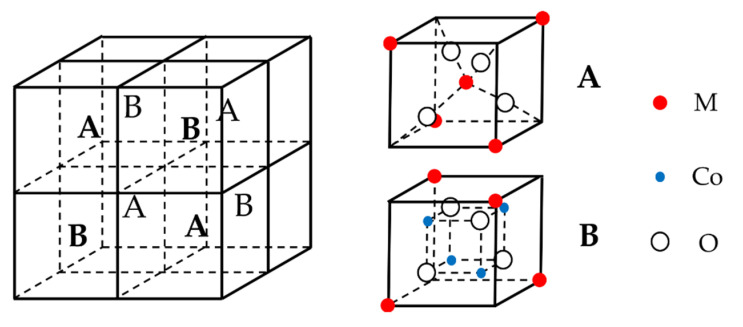
Schematic crystal structure of MCo_2_O_4_.

Cobalt-containing ternary metal oxides are typical spinel structures, and the cells of spinel consist of eight small cubic cells, which are four A-type cells and four B-type cells interconnected (Figure 4). Each A-type or B-type unit has four O^2−^ for a total of 32. M ions are in the center of the A-type unit (tetrahedral gap) and half of the vertices of the eight small cubic units for a total of eight. Cobalt ions occupy each of four octahedral gaps, for a total of 16. The cell general formula of cobalt-based spinel is M_8_Co_16_O_32_, and the chemical formula is summarized as MCo_2_O_4_. Furthermore, in general, the alkaline electrolytes of different Co-based spinel MCo_2_O_4_ (M = Co, Ni, Fe, and Mn) undergo approximately the same reversible electrochemical redox reactions with the discharge products of M ions as hydroxyl oxides MOOH [72,73,74]. The resulting MOOH (M = Co, Fe, and Mn) further discharges and produces the corresponding CoO_2_ [72], FeO_4_^2−^ [75] and MnO_2_. Because of the presence of Cu(I)/Cu(II) pairs, the discharge products of Zn^2+^ and Cu^2+^ are Zn(II) [76] and Cu(I)/Cu(II) [77] hydroxides.

Metal oxides with multiple metal cations generally have higher conductivity and capacitive activity than single metal oxides [78]. Among them, ternary transition metal oxides provide more active sites for redox reaction and improve electronic conductivity because they have two different cations [79]. Compared with binary transition metal oxides such as Co_3_O_4_, the electrochemical properties of ternary transition metal oxides (MnCo_2_O_4_, NiCo_2_O_4_, ZnCo_2_O_4_, etc.) are significantly improved under the influence of the synergistic effect generated by the coupling of two transition metals [36].

#### 2.3.1. MnCo_2_O_4_

MnCo_2_O_4_ is a typical compound with a spinel structure. It can show two lattice structures: (a) normal spinel [80,81], (b) anti spinel [82]. Due to the diversity of crystal structure, the variation of charges (Mn and Co) occupied in octahedron and tetrahedron makes it have excellent redox stability [83]. Manganese transmits more electrons and has higher capacity, while cobalt has higher oxidation potential. Many experiments have proved that MnCo_2_O_4_ improves the electrochemical performance of single Co_3_O_4_ and shows better conductivity, structural stability, and cycle performance [84,85,86]. The reaction principle of MnCo_2_O_4_ is as follows:

MnCo_2_O_4_ + OH^−^ + H_2_O → MnOOH + 2CoOOH + e^−^ (3)

MnOOH + OH^−^ → MnO_2_ + H_2_O + e^−^ (4)

CoOOH + OH^−^ → CoO_2_ + H_2_O + e^−^ (5)

MnCo_2_O_4_ reacts under alkaline conditions to form MnOOH and CoOOH, and the resulting MnOOH and CoOOH continue to react with OH^−^ to form MnO_2_ and CoO_2_, while releasing electrons.

**Figure 5:**
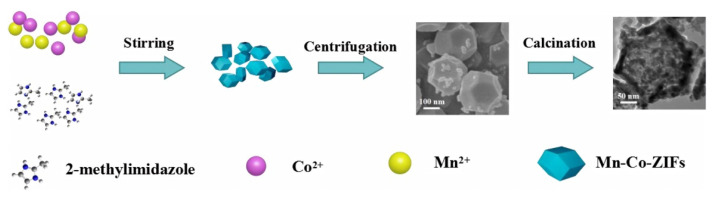
Preparation process of a nanocage MnCo_2_O_4_ electrode. Reproduced with permission from Yanying Dong, Facile synthesis of hierarchical nanocage MnCo_2_O_4_ for high performance supercapacitor; published by Elsevier, 2016 [89].

**Figure 6:**
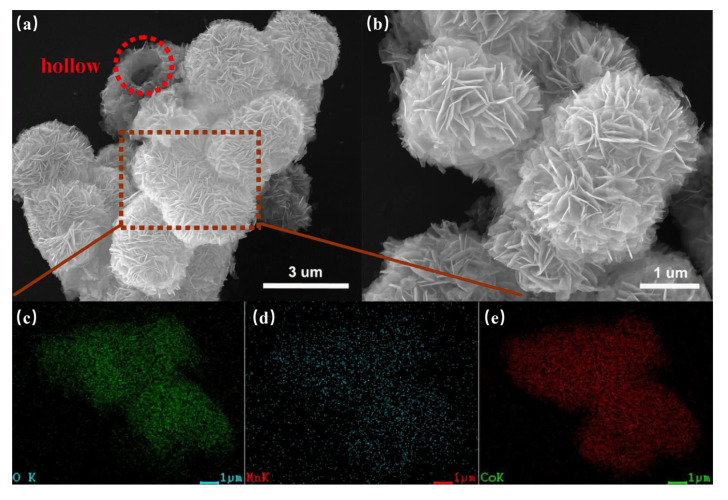
SEM images (**a**,**b**) of the calcined MnCo_2_O_4_ products and the corresponding elements mapping (**c**–**e**) taken from the square area marked in Figure 6a. Reproduced with permission from Hongwei Che, Template-free synthesis of novel flower-like MnCo_2_O_4_ hollow microspheres for application in supercapacitors; published by Elsevier, 2016 [87].

Based on the above studies, MnCo_2_O_4_ is considered an ideal candidate material for SCs, so it has been widely studied. Various forms of MnCo_2_O_4_ materials have been prepared, such as flower shaped hollow microspheres [87], core-shell structures [88], nano cages [89], nano needles [90], ellipsoids [91], and sea urchins [92]. For example, Dong et al. synthesized MnCo_2_O_4_ with a hierarchical nanocage structure using a bimetallic zeolite imidazolate framework as the precursor and template [89]. The preparation process and morphological characterization are shown in Figure 5. Through the analysis of its micro morphology, it can be found that many interconnected nanoparticles form a highly porous nanocage structure. This unique nanocage structure exposes a large area of surface and mesoporous structure, which promotes the diffusion of ions and ensures its excellent electrochemical performance in SCs. By testing the electrochemical performance of MnCo_2_O_4_ electrode, it was found that it can show 95% capacitance retention after 4500 cycles at 1 A g^−1^, which proves its superior cycle stability. Che et al. synthesized flower-shaped MnCo_2_O_4_ hollow microspheres with a nano flower structure by the template free method of mixing and heating the solvent to 180 °C [87], and then calcining at 350 °C for two hours. An SEM microscopic image is shown in the Figure 6. The larger surface area and porous structure provide more active sites, promote the transfer of ions and electrons, accelerate the reaction rate, and greatly enhance its electrochemical storage performance. The capacity retention rate of the electrode was 93.6% after 2000 consecutive cycles at a high current density of 1 A g^−1^.

Although the electrochemical performance of an MnCo_2_O_4_ electrode is significantly improved compared with a single Co_3_O_4_ electrode, its development is limited by its poor cycle stability in long-term use. To solve this problem, one of the effective methods is to compound it with some carbonaceous material with light electric power or other pseudo-capacitive oxide or hydroxide to improve its cycle stability. For example, Wang et al. synthesized a 3D porous structure based on MnCo_2_O_4_ modified graphene [93]. The specific capacitance reached 503 F g^−1^ at a current density of 1 A g^−1^. After 5000 charge-discharge cycles (current density of 10 A g^−1^), 97.4% of the specific capacitance was retained.

**Figure 7:**
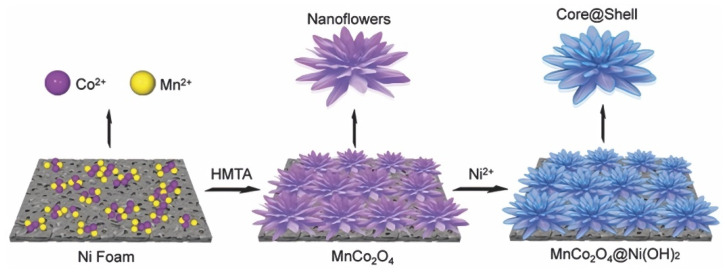
Schematic illustration of the general electrode design process. Reproduced with permission from Limin Wu, Preparation of MnCo_2_O_4_@Ni(OH)_2_ Core-Shell Flowers for Asymmetric Supercapacitor Materials with Ultrahigh Specific Capacitance; published by Wiley-VCH Verlag, 2016 [88].

Zhao et al. synthesized an MnCo_2_O_4_@Ni(OH)_2_ multicomponent composite by a stepwise hydrothermal method [88]. The synthesis process is shown in Figure 7. First, layered double hydroxides of cobalt and manganese were generated with hexamethylenetetramine as a structure guide. With the directional attachment process as the driving force, the nanoparticles finally grew into MnCo_2_O_4_ nanostructures. Then, using nickel chloride and hexamethylenetetramine as the lead solution, an ultra-thin Ni(OH)_2_ nano sheet was fixed on the nano alloy by hydrothermal method to produce a layered MnCo_2_O_4_@Ni(OH)_2_ core-shell structure. The discharge time of MnCo_2_O_4_@Ni(OH)_2_ was about four times that of MnCo_2_O_4_.

The specific capacitance of activated carbon electrode can reach 328 F g^−1^ at 0.2 A g^−1^, and the maximum energy density of asymmetric SC (ASC) can reach 48 W h kg^−1^ when the mean power density is 1.4 kW kg^−1^, which is significantly higher than that of most commercial batteries. In addition, the capacitance retention of the hybrid electrode is about 90% after 2500 cycles at a current density of 6 A g^−1^, and the structure of the nano alloy remains good. The above results show that the electrochemical performance of MnCo_2_O_4_ is significantly improved and its cycle stability is higher by compounding MnCo_2_O_4_ with Ni(OH)_2_.

As mentioned above, compared with a single MnCo_2_O_4_ material, MnCo_2_O_4_ compounded with other materials has higher cycle stability and greater prospects. Although the electrochemistry of the material can be improved to some extent by changing the morphology and structure or compounding with other materials, the low conductivity of MnCo_2_O_4_ has hindered its wide application as an energy storage device. At the same time, how to accurately control the micro morphology of the composite still needs further exploration.

#### 2.3.2. NiCo_2_O_4_

As a typical cobalt-containing ternary metal oxide, NiCo_2_O_4_ is also a transition metal oxide with a spinel structure. It has the advantages of high electrochemical activity, good conductivity, high theoretical capacitance, low cost, and simple synthesis. Therefore, NiCo_2_O_4_ is also one of the most attractive electrode materials in SCs [94,95,96,97,98]. In its structure, nickel ions occupy octahedral sites, and cobalt ions diffuse in octahedral and tetrahedral sites [99]. The electronic conductivity and electrochemical activity of NiCo_2_O_4_ are significantly higher than those of nickel oxide and cobalt oxide alone due to the synergistic effect of Ni with Co.

At present, various nanostructures of NiCo_2_O_4_ have been prepared, such as nanowires [100], nanosheets [101], nanoflowers [102], and nanorods [103]. Among them, hollow nano materials have a large surface area, large gap and short effective transmission distance of electrolyte ions [104]. They provide more electroactive sites for rapid ion insertion of the whole electrode material and show excellent electrochemical performance. Xu et al. synthesized hollow NiCo_2_O_4_ nanospheres with a layered structure [104]. When using them as electrodes, the specific capacitance at 1 A g^−1^ is 1229 F g^−1^, which is higher than that of NiCo_2_S_4_ hollow spheres (1036 F g^−1^ at 1 A g^−1^) [105], NiCo_2_O_4_ hollow spheres (1141 F g^−1^ at 1 A g^−1^) [106], hollow NiCo_2_O_4_ sub microspheres (678 F g^−1^ at 1 A g^−1^) [107], urchin-like NiCo_2_O_4_ hollow microspheres (942.2 F g^−1^ at 0.5 A g^−1^) [108], and mesoporous NiCo_2_O_4_ hollow microspheres (987 F g^−1^ at 1 A g^−1^) [109]. After 3000 cycles at 50 mV s^−1^, the total specific capacitance retention of hollow NiCo_2_O_4_ nanosphere electrode is 86.3%, while the total specific capacitance retention of NiCo_2_O_4_ microsphere electrode is 83.7%.

Although hollow microspheres can effectively improve surface area, the single-structure NiCo_2_O_4_ electrode material still has the disadvantages of low conductivity, limited kinetics, and poor electrochemical performance [110,111,112,113,114]. To improve its electrochemical performance, constructing NiCo_2_O_4_ layered nanostructure composites has become an important means [115,116,117,118,119]. For example, Zhou and others synthesized 3D porous graphene/NiCo_2_O_4_ hybrid films with copper oxide as a template [120]. Its unique 3D porous structure can store many electrolytes and provide rich active centers, thus improving the electrochemical performance. At 1 A g^−1^, the specific capacitance can reach 708.36 F g^−1^. After 6000 cycles at 10 A g^−1^, the initial capacitance of 94.3% is maintained. Li et al. prepared flower-like hollow C@MnCo_2_O_4_ with high specific surface area. At a discharge current density of 1 A g^−1^, the discharge capacitance reached 728.4 F g^−1^, and after 1000 cycles at 8 A g^−1^ the initial capacitance retention of the composite was 95.9% [121]. Zhao et al. synthesized ultra-thin NiCo_2_O_4_/NiO nanosheets grown on silicon nitride [122]. After 2000 cycles at 20 mA cm^−2^ current density, the specific capacitance retention was 90.9%, and the energy density was 60 W h kg^−1^ when the power density was 1.66 kW kg^−1^. Cheng et al. prepared a 3D layered NiCo_2_O_4_@NiMoO_4_ nuclear shell nanowires/nanowire sheet array on nickel foam, with a capacitance retention rate of 85.2% after 3000 cycles at a current density of 20 mA cm^−2^ [123]. After a long cycle, the volume resistance of the ASC device increased slightly from the initial 0.40 Ω to 0.42 Ω. The above shows that the prepared composites have good cycle stability. Lee et al. synthesized MnCo_2_O_4_-NiCo_2_O_4_ composite with layered nanostructure by one-step chemical bath deposition method [124]. When used as an electrode, the specific capacitance reached 1152 F g^−1^ at 1 A g^−1^. After 3000 cycles at 6 A g^−1^, the specific capacitance retention of the composite was 95.38%, while NiCo_2_O_4_ is 86.14% and MnCo_2_O_4_ was 61.65%, indicating that the composite of the two materials significantly increased the cycle stability of the material.

**Figure 8:**
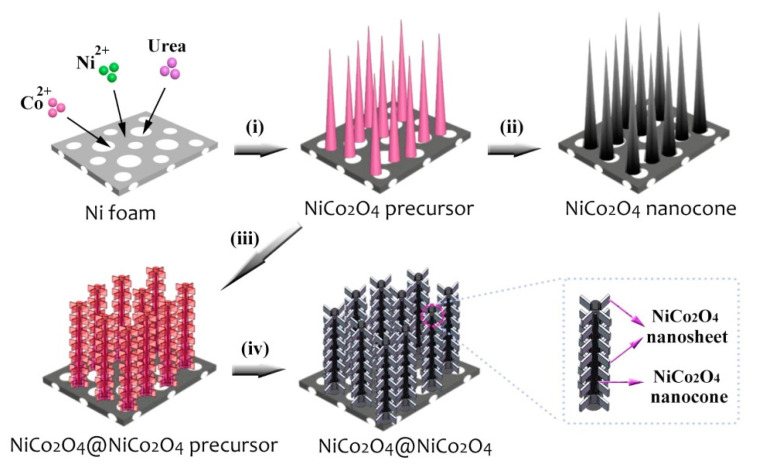
Schematic of the fabrication process for 3D NiCo_2_O_4_@NiCo_2_O_4_ hierarchical core-shell NCAs on Ni foam. Reproduced with permission from Xiuhua Wang, Three-Dimensional NiCo_2_O_4_@NiCo_2_O_4_ Core-Shell Nanocones Arrays for High-Performance Supercapacitors; published by Elsevier, 2018 [94].

The electrochemical properties of the above NiCo_2_O_4_ composite have been significantly improved. However, due to lattice mismatch between NiCo_2_O_4_ and other components, this leads to poor structural stability, lower specific capacitance and cycle life. Therefore, Wang et al. compounded NiCo_2_O_4_ and NiCo_2_O_4_ with the same lattice type to prepare 3D delamination NiCo_2_O_4_@NiCo_2_O_4_ [94]. The preparation process of the core-shell nano cone array is shown in Figure 8. First, NiCo_2_O_4_ is grown vertically on nickel foam by hydrothermal method. After annealing, neat NiCo_2_O_4_ nano-cone arrays is formed first. Then, the NiCo_2_O_4_ nanosheet is coated on the NiCo_2_O_4_ surface formed in the previous step. Finally, layered core-shell NiCo_2_O_4_@NiCo_2_O_4_ nanostructures are fabricated on nickel foams after subsequent annealing.

After 21,000 cycles at 4 A g^−1^, the capacitance retention rate of the electrode reached 85.3%, and the structure did not change significantly during charge and discharge. When used in SCs, NiCo_2_O_4_@NiCo_2_O_4_ core-shell nanostructure had a capacitance of 2045.2 F g^−1^ at a current density of 1 A g^−1^, which is better than the single component of NiCo_2_O_4_ nanosheet (346.4 F g^−1^) and NiCo_2_O_4_ nano cone (1381.8 F g^−1^).

#### 2.3.3. ZnCo_2_O_4_

Similar to MnCo_2_O_4_ and NiCo_2_O_4_ mentioned above, ZnCo_2_O_4_ has the advantages of high theoretical capacitance, high conductivity, environmental friendliness, and low cost, and is considered as a potential SC material [125]. At present, ZnCo_2_O_4_ materials with various nanostructures, such as nanowires [126], nanosheets [127,128] nanoparticles [129], and nanospheres [130,131], have been prepared. For example, Wang and colleagues synthesized ZnCo_2_O_4_ nanowire electrode materials grown on nickel foam [132]. First, the precursor ZnCo_2_O_4_ nanowire arrays were grown on nickel foam by a hydrothermal reaction and then calcined in air. Finally, ZnCo_2_O_4_ nanowire arrays supported by nickel foam were obtained. The synthesized ZnCo_2_O_4_ nanowires have a porous structure, which makes the material have large specific surface area and can promote the diffusion of reactants. The prepared ZnCo_2_O_4_ nanowire/nickel foam electrode had a specific capacitance of 1625 F g^−1^ at a current density of 5 A g^−1^, and 94% of the original capacitance was maintained after 5000 cycles at 20 A g^−1^.

**Figure 9:**
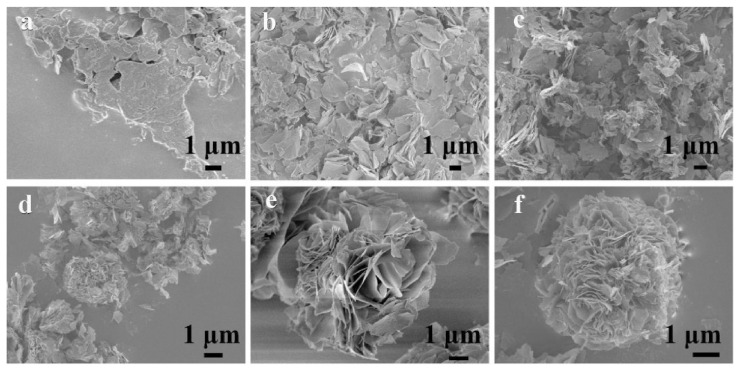
SEM images of ZnCo-preprepared with different reaction times: (**a**) 4 h, (**b**) 6 h, (**c**) 12 h, (**d**) 18 h, (**e**) 21 h, and (**f**) 24 h. Reproduced with permission from Liangyu Shang, Self-assembled hierarchical peony-like ZnCo_2_O_4_ for high-performance asymmetric supercapacitors; published by Elsevier, 2017 [135].

Xu et al. prepared a ZnCo_2_O_4_ nanostructure with a porous structure and found that the conversion between nanosheets and nanowires was obtained by regulating hydrothermal temperature [133]. When the current density was 1 A g^−1^, ZnCo_2_O_4_ had a specific capacitance of 776.2 F g^−1^, and the energy density was 84.48 W h kg^−1^ when the mean power density was 0.4 kW kg^−1^. It had 84.3% capacity retention after 1500 cycles (3 A g^−1^). Venkatachalam et al. prepared hexagonal-like ZnCo_2_O_4_ nanomaterials by a simple hydrothermal method [134]. The prepared electrode materials had a specific capacitance of 845.7 F g^−1^ at a current density of 1 A g^−1^, and retained 95.3% of the original capacitance after 5000 cycles at 5 A g^−1^. Shang et al. synthesized 3D layered peony flower-like ZnCo_2_O_4_ electrode nanomaterials by a simple solvothermal method and annealing without additives [135]. The microstructure is shown in Figure 9. The assembled ASC ZnCo_2_O_4_//active carbon had an energy density of 29.76 W h kg^−1^ at a power density of 398.53 W kg^−1^. In addition, the peony-shaped ZnCo_2_O_4_ electrode material had a specific capacitance of 440 F g^−1^ at a current density of 1 A g^−1^, and the capacitance was maintained at 155.6% after 3000 cycles (2 A g^−1^).

Although the above nano ZnCo_2_O_4_ materials have specific applications in SCs, the insufficient utilization efficiency and poor conductivity of the materials limit their electrochemical properties to a certain extent and there are difficulties in them meeting the needs of practical applications. To solve this problem, one of the commonly used methods is to introduce oxygen vacancies. The existence of an oxygen vacancy can significantly improve the conductivity of ZnCo_2_O_4_, adjust the electronic structure, increase the active sites, and promote the electrochemical performance of SCs. For example, Xiang and his colleagues prepared two-dimensional (2D) ZnCo_2_O_4_ nanosheets rich in oxygen vacancies [136]. The nanoscale thickness and large surface area effectively improved the utilization of the electrode while promoting electron transfer. A specific capacitance of 2111 F g^−1^ was attained at a current density of 1 A g^−1^, while the specific capacitance of the original ZnCo_2_O_4_ nano sheet at the same current density was only 1121 F g^−1^. When the power density was 160 W kg^−1^, the energy density of ASC constructed by ZnCo_2_O_4_ nanosheet (with oxygen vacancy)//activated carbon is 34.6 W h kg^−1^, and 93% of the original capacitance was maintained after 3000 cycles at 2 A g^−1^.

**Figure 10:**
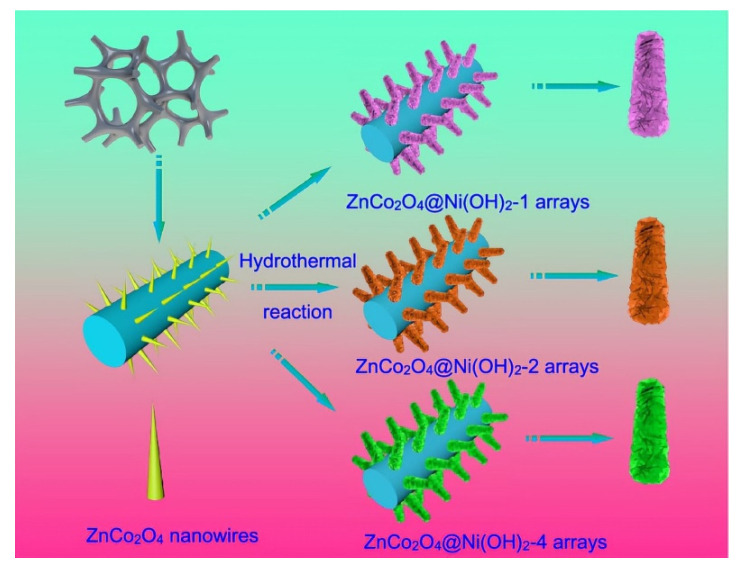
Synthesis schematic of synthesized ZnCo_2_O_4_@Ni(OH)_2_ samples. Reproduced with permission from Meizhen Dai, Ni Foam Substrates Modified with a ZnCo_2_O_4_ Nanowire-Coated Ni(OH)_2_ Nanosheet Electrode for Hybrid Capacitors and Electrocatalysts; published by ACS Publications, 2021 [137].

Combining ZnCo_2_O_4_ nanostructures with conductive metal or carbon materials to construct composites is one of the methods to alleviate the above problems. For example, Wu et al. synthesized a series of ZnCo_2_O_4_@Ni(OH)_2_ nanostructures grown on nickel foam by a two-step hydrothermal method; the preparation process is shown in Figure 10 [137]. First, the ZnCo_2_O_4_ nanowires were uniformly covered on the nickel foam by a hydrothermal method and then Ni(OH)_2_ nanosheets were grown on the ZnCo_2_O_4_ nanowire after a second hydrothermal reaction. ZnCo_2_O_4_ nanowires were used as the substrate and Ni(OH)_2_ nanosheets were used as the upper layer. The strong binding force between them reduced the contact resistance and promote the transfer of electrons to enhance the electrochemical reaction activity of the material. The synthesized hybrid structure was used to fabricate capacitors with an energy density of 57.3 W kg^−1^ at 4675.3 W h kg^−1^, and an initial capacitance of 48.6 C g^−1^ at 1 A g^−1^, which retained 90.5% after 10,000 cycles at the same current density.

**Figure 11:**
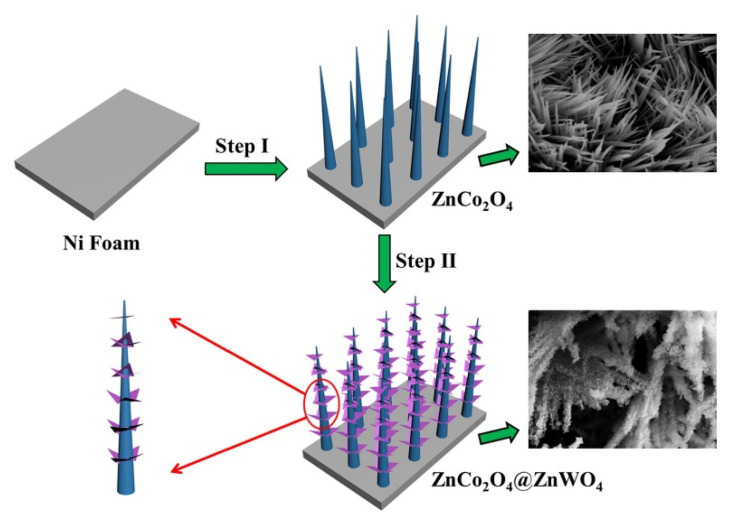
Schematic illustrating the fabrication process of the ZnCo_2_O_4_@ZnWO_4_ core-shell nanowire arrays on a nickel foam substrate. Reproduced with permission from Li Xie, Core-shell structured ZnCo_2_O_4_@ZnWO_4_ nanowire arrays on nickel foam for advanced asymmetric supercapacitors; published by Elsevier, 2018 [138].

Xie et al. synthesized a ZnCo_2_O_4_@ZnWO_4_ nanowire array with a core-shell structure on nickel foam, and the synergistic effect between ZnCo_2_O_4_ nanowire and ZnWO_4_ sheet effectively improved the electrochemical performance of hybrid electrode [138]. The synthesis process is like that of ZnCo_2_O_4_@Ni(OH)_2_. As shown in Figure 11, ZnCo_2_O_4_ nanowires are first grown on nickel foam, and then ZnWO_4_ nanosheets arrays are produced by a simple hydrothermal method using ZnCo_2_O_4_ nanowires as skeletons. The constructed ZnCo_2_O_4_@ZnWO_4_//active carbon ASC had an energy density of 24 W h kg^−1^ at a power density of 400 W kg^−1^. The original capacitance retention was 98.5% after 5000 cycles at a current density of 100 mA cm^−2^.

Table 2 summarizes the structure, specific capacity and cycling performance of MnCo_2_O_4_, NiCo_2_O_4_ and ZnCo_2_O_4_. The electrochemical performance of the electrodes was significantly improved after designing unique morphologies for the materials. By constructing effective structures, such as spherical, rod-like, and hollow structures, the contact area can be increased, resulting in close contact between the active material and the electrolyte, which leads to high electrochemical activity and enhanced pseudocapacitive performance. In addition, the porous structure can alleviate the volume change caused by the redox reaction, thus improving the cycle performance, so the materials in the table are often designed as porous structures. Among the various unique morphologies, 2D microstructures are an important category because such structures increase the contact area between the electrolyte and the electrode material. For example, Younis et al. designed various micro morphologies including nanowires, nanocables, nano-micro biscuits, and micro-walls [139]. Among them, nano-micro biscuits with distinct 2D structural features exhibited the best electrochemical performance. Xiang et al. designed ZnCo_2_O_4_ nanosheets with nanoscale thickness and large surface area, which could improve the electron transfer efficiency and electrode utilization [136]. In addition, Zhang et al. prepared NiCo_2_O_4_ nanosheets with a more ordered crystal structure, high specific surface area and diffusion channels [140]. Liu et al. prepared MnCo_2_O_4_ with a nanoflower-like morphology and porous structure [141]. Because of its unique nanostructure, the prepared electrode had high capacity and good rate performance. In conclusion, 2D nanostructures usually have a large surface area and dense porous structure. This structure is beneficial to increase the contact area between electrolyte and electrode, thus improving the electron transfer efficiency.

### 2.4. Cobalt-Containing Ternary Metal Oxide Derivatives

As mentioned above, cobalt-containing ternary metal oxides have great potential in the application of SCs. To further improve their electrochemical performance, researchers have focused on the derivatives of these metal oxides. Transition metal sulfides have high electronic conductivity, two orders of magnitude higher than the corresponding oxides, because the valence states of the transition metals in the sulfides closely resemble those of the metals [142,143,144]. At the same time, because sulfur is less electronegative than oxygen, it can produce a more flexible structure instead of oxygen. This can effectively avoid the structural disintegration of transition metal sulfide-based electrodes due to interlayer elongation, which facilitates the transport of electrons in the internal structure [145]. In addition, combining two or more sulfides can improve the electrical properties of transition metal sulfides, resulting in a richer redox reaction [146,147,148] because bimetallic sulfides possess more prosperous diverse states, smaller optical band gaps, and better chemical stability than single-metal sulfides [144,148]. Compared with single metal oxide, transition metal sulfides such as Co-Mo-S, NiCo_2_S_4_ have higher capacitance, multivalent redox reactions and higher conductivity [149], so they have great potential.

#### 2.4.1. Co-Mo-S

Co-Mo-S matrix composites have great potential as SC electrode materials because of their advantages of reversible redox reaction band gap, high conductivity, and low electronegativity [149,150,151,152,153,154,155,156,157,158,159]. For example, Balamurugan et al. used ion exchange reaction technology to synthesize a porous nano foam support structure composed of ultra-thin Co-Mo-S nanosheets [160]. When Co-Mo-S nanosheets are used as the electrode of the SC, they can provide an ultra-high specific capacitance of 2343 F g^−1^ at a current density of 1 mA cm^−2^, and the capacitance remains 96.6% after 20,000 cycles. In addition, the energy density and power density of Co-Mo-S/nitrogen doped graphene nanosheets assembled in ASC are 89.6 W h kg^−1^ and 20.07 W kg^−1^. The capacitance retention rate can reach 86.8% after 50,000 cycles. The unique electrochemical properties of Co-Mo-S nanosheets are attributed to the ultra-high contact area with 3D nickel foam and electrolyte.

**Figure 12:**
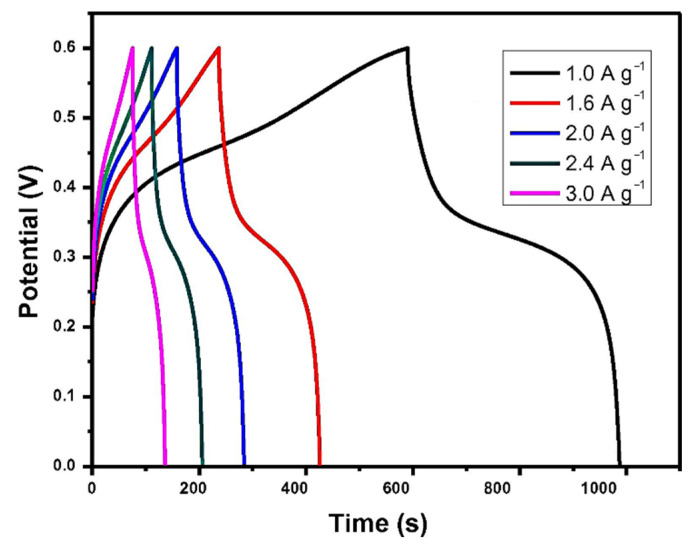
Galvanostatic charge/discharge curves of CoMoS_4_. Reproduced with permission from Xiaoyang Xu, Amorphous CoMoS_4_ for a valuable energy storage material candidate; published by Elsevier, 2016 [161].

Xu et al. prepared amorphous CoMoS_4_ by a simple precipitation method and used it as an SC material for the first time [161]. Changing the current density from 1 A g^−1^ to 3 A g^−1^, the galvanostatic charge/discharge curves are shown in Figure 12 when the potential is from 0 V to 0.6 V. The specific capacitance was calculated according to these curves. The results show that it had a specific capacitance of 661 F g^−1^ at a current density of 1 A g^−1^. Simultaneously, the constructed CoMoS_4_//reduced graphene oxide hybrid SC had a particular capacity of 77 F g^−1^ at a current density of 0.5 A g^−1^, and its energy density was 27.2 W h kg^−1^ at a power density of 400 W kg^−1^. In addition, after 10,000 cycles at 80 mV s^−1^, the original capacitance was maintained at about 86%.

Recently, Sun et al. synthesized Co-Mo-S nanosheet networks by a simple two-step hydrothermal method [162]. The ASC assembled with the product as the cathode had an energy density of 72.25 W h kg^−1^ at 2700 W kg^−1^. After 9000 cycles at 2 A g^−1^, the capacitance retention rate reached 83.4%.

**Figure 13:**
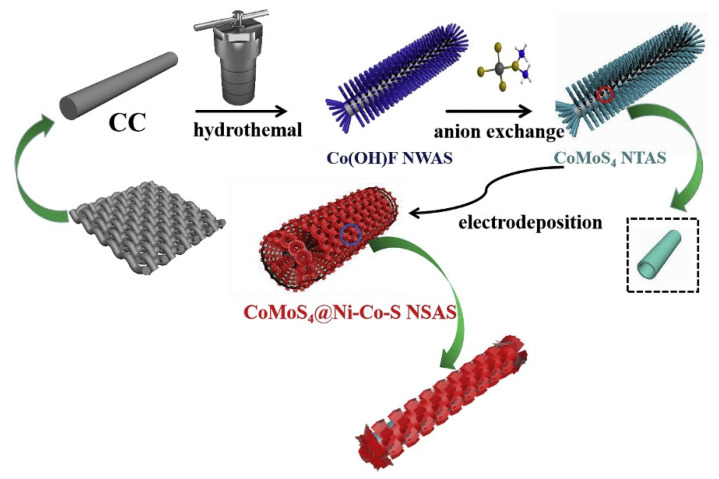
Schematic illustration of the fabrication of a hierarchical core-shell hollow CoMoS_4_@Ni-Co-S nanotubes electrode. Reproduced with permission from Fei Ma, Hierarchical core-shell hollow CoMoS_4_@Ni-Co-S nanotubes hybrid arrays as advanced electrode material for supercapacitors; published by Elsevier, 2019 [163].

Although Co-Mo-S has excellent potential in SCs, its relatively poor rate capability and cycle stability limit its application. Overcoming these disadvantages and improve its electrochemical properties has become a key problem of Co-Mo-S capacitor materials. A practical method is to achieve excellent cycle capacity and rate performance by construction of the electrode material structure. Ma et al. designed and constructed hollow core-shell CoMoS_4_@Ni-Co-S nanotubes on carbon cloth for the first time by a hydrothermal method and electrodeposition process [163]. The preparation process of Co-S nanotubes is shown in Figure 13. First, Co(OH)F nanowire arrays are synthesized by hydrothermal reaction under high temperature and high pressure with carbon cloth as a current collector. Then, Co(OH)F nanowires and (NH_4_)_2_MoS_4_ precursor solution ae transformed into CoMoS_4_ nanotubes. Finally, 3D layered CoMoS_4_@Ni-Co-S nanotube hybrid arrays are synthesized by electrochemical deposition method. Among them, Ni-Co-S nanosheets are closely arranged around CoMoS_4_ hollow nanotubes, which is conducive to the exposure of electrochemical active sites and keeps the structure stable to a certain extent during charge and discharge. At the same time, the core-shell structure facilitates the close contact of the electrode/electrolyte and avoids the aggregation of Ni-Co-S. The novel CoMoS_4_@Ni-Co-S electrode had an excellent specific capacitance of 2208.5 F g^−1^ at 1 A g^−1^ and good cycle life (91.3% capacitance retention over 5000 cycles at 3 A g^−1^). In addition, the assembled CoMoS_4_@Ni-Co-S//activated carbon ASC had an energy density of 49.1 W h kg^−1^ at 800 W kg^−1^ and a capacity retention rate of 90.3% after 10,000 cycles.

#### 2.4.2. NiCo_2_S_4_

As mentioned earlier, NiCo_2_O_4_ and its composites have great potential in SCs. The conductivity of NiCo_2_S_4_ is 100 times that of NiCo_2_O_4_, and NiCo_2_S_4_ shows higher electrochemical activity and capacitance than other cobalt nickel compounds because of its inherent redox reaction center. However, NiCo_2_S_4_-based electrodes suffer from defects such as easy oxidation in alkaline electrolytes and poor long-term cycling stability [164]. Therefore, effective space structures need to be designed to improve their drawbacks. So far, 3D NiCo_2_S_4_ nanostructures such as nanoflowers, core-shell and dendrites have been synthesized.

For example, Shi et al. synthesized layered sea urchin-like hollow NiCo_2_S_4_ by a template-free solvothermal method [165]. The capacitance reached 1398 F g^−1^ at 1 A g^−1^, and the specific capacity retention rate reached 74.4% after 5000 cycles at 10 A g^−1^. Zhang et al. synthesized nano NiCo_2_S_4_ with 3D honeycomb structure by a hydrothermal method and vulcanization method [166]. When the current density was 1 mA cm^−2^, its maximum specific capacity exceeded 14 mA h cm^−2^. After 1000 cycles at a current density of 10 mA cm^−2^, the specific capacity remained at 96.96%.

These structures have been widely used in electrode materials. However, their poor electronic conductivity and potential risk of structural collapse and damage during long-term use limit the application of NiCo_2_S_4_ materials. One of the main methods to solve this problem is to build 3D hierarchical structure materials and increase the contact area with electrolyte.

Li et al. successfully synthesized layered dendritic NiCo_2_S_4_@NiCo_2_S by a three-step continuous hydrothermal method, and the layered microstructure of the highly porous structure facilitated ion transport during charge and discharge, resulting in a significant improvement in electrochemical performance. When the current density was 240 mA cm^−2^, the electrode discharge specific capacity of the dendritic structure reached 4.43 mA h cm^−2^. When the current density was increased from 40 mA cm^−2^ to 240 mA cm^−2^, its rate capability reached 70.1% [167]. Tang et al. synthesized ultra-high load (10.33 mA cm^−2^) 3D layered NiCo_2_S_4_/Ni_3_S_2_ nanosheets with an energy density of 4.69 W h m^−2^ (power density of 10.33 W m^−2^), and a stability of 91.4% after 8000 cycles at 20.66 mA cm^−2^ [168].

Zhang et al. synthesized NiCo_2_S_4_ spheres with granular nuclei by a simple two-step hydrothermal reaction [169]. A NiCo_2_(OH)_6_/C precursor was prepared using a carbon pellet cluster as a template. Granular NiCo_2_S_4_ was synthesized by reacting with sodium sulfide, and then the NiCo_2_S_4_ precursor was grown on the periphery of the granular NiCo_2_S_4_ to form a unique structure. The specific surface area of the prepared NiCo_2_S_4_ ball was 26.61 m^2^ g^−1^, which is about twice that of the particle NiCo_2_S_4_ (11.41 m^2^ g^−1^). This higher specific surface area increased the electroactive sites that can transfer charge and shortens the transmission path, which is conducive to improving the electrochemical activity of the material. When the current density was 1 A g^−1^, the specific capacitance of the granular NiCo_2_S_4_ spherical electrode reached 1156 F g^−1^, which was 71% higher than that of the NiCo_2_S_4_ electrode. In addition, after 1000 charge-discharge cycles (5 A g^−1^), the NiCo_2_S_4_ sphere electrode with granular nuclear showed 82% capacitance retention, and the cycle stability was significantly better than that of the granular NiCo_2_S_4_ electrode.

**Figure 14:**
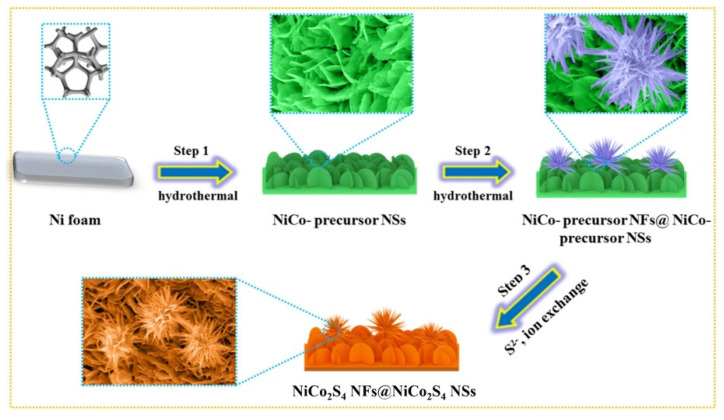
Schematic diagram showing the fabrication of NiCo_2_S_4_ nanoflowers @NiCo_2_S_4_ nanosheets. Reproduced with permission from Wenling Wu, Hierarchical structure of Self-Supported NiCo_2_S_4_ Nanoflowers@NiCo_2_S_4_ nanosheets as high rate-capability and cycling-stability electrodes for advanced supercapacitor; published by Elsevier, 2021 [170].

Wu et al. prepared a hierarchical nanostructured NiCo_2_S_4_ nanoflower@NiCo_2_S_4_ nanosheet material by a hydrothermal method (Figure 14) [170]. Using this composite as the electrode in the SC, it had a specific capacity of 338.1 mA h g^−1^ at 2 mV s^−1^, which is about three times higher than that of a single NiCo_2_S_4_ nanosheet. In addition, 90% of the original capacity was maintained after 4000 reaction cycles at a current density of 20 A g^−1^. The synthesized NiCo_2_S_4_ nanoflowers@NiCo_2_S_4_ nanosheets//NiCo_2_S_4_ nanoflowers@NiCo_2_S_4_ nanosheets symmetrical SC device had an energy density of 18.05 W h kg^−1^ at a power density of 750 W kg^−1^. The capacitance retention rate of the symmetrical SC device was 89% after 4000 cycles (10 A g^−1^). The multilayer 3D structure can explain this improvement in electrochemical performance. The upper nanoflowers are composed of many rough nanotubes, which increase the surface volume ratio and the contact range of the electrolyte. This unique structure can provide more electrochemical active sites, promote ion adsorption, and reduce the volume expansion in the charge and discharge process. Furthermore, the lower layer nanosheet arrays on the nickel foam can avoid damage and increase the stability of the electrochemical reaction.

Densely arranged and structurally stable nanosheets can act as a charge transport interconnectors with nickel foam, further improving the charge transport rate. This unique synergistic effect between nanoflower and nanosheet structure effectively increases the structural stability and electrochemical active sites of the material. The effect also promotes charge transfer and ion transport, which is conducive to accelerating the electrochemical reaction rate and improving the energy storage effect of the material.

As mentioned above, the construction of 3D multilayer hierarchical structures can improve the electrochemical properties of materials and in-use stability. At the same time, the construction of nanostructured composites by doping other impurity atoms is the foremost solution to the problem of low electronic conductivity and poor stability of NiCo_2_S_4_ materials. Among them, carbon material has superior conductivity [170], which can promote charge transfer. Due to the strong coupling between the carbon substrate and metal-based oxide, the composite of carbon material and NiCo_2_S_4_ can effectively increase the electrochemical activity of electrode material [171]. For example, Pezzotti and co-workers synthesized a kelp-like NiCo_2_S_4_-C-MoS_2_ composite by hydrothermal and solvothermal methods [172]. It had a specific capacitance of 1601 F g^−1^ at a current density of 0.5 A g^−1^ and 75% of the initial specific capacity after 2000 cycles at a current density of 2 A g^−1^. Shim et al. synthesized a hollow C-NiCo_2_S_4_ nano-lake sheet structure with a one-step solvent method [173]. The specific capacitance reached 1722 F g^−1^ at a current density of 1 A g^−1^, and 95.60% capacity retention after 5000 cycles at a current density of 10 A g^−1^.

In addition, since the electronegativity and atomic radius of P and S atoms are similar, introducing P atoms results in lattice distortion, providing more active sites. Therefore, the introduction of the P atom is also a way to improve the electrochemical activity of materials. Based on the above, Liu et al. introduced P and C elements into a NiCo_2_S_4_ electrode material by a one-step solvothermal method and phosphating process [174]. As the electrode material of SCs, it had a specific capacity of 1026 C g^−1^ at a current density of 1 A g^−1^, and an original capacity retention rate of 89% after 20,000 cycles at 10 A g^−1^. In comparison, NiCo_2_S_4_ only reached 65% of the original capacity under the same conditions. The ASC had an energy density of 131.40 W h kg^−1^ at a power density of 1355.37 W kg^−1^, and 96.3% of the original capacity was maintained after 10,000 cycles at a current density of 2 A g^−1^.

**Figure 15:**
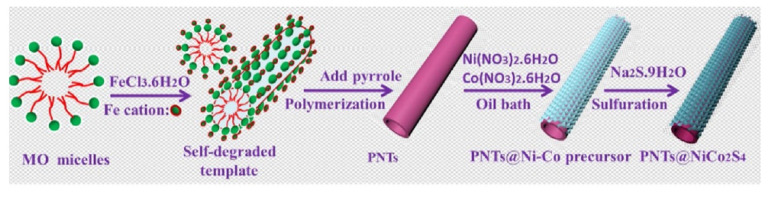
Schematic illustration of polyporrole nanotubes@NiCo_2_S_4_ core-shell formation. Reproduced with permission from Jun Zhang, Hierarchical polypyrrole nanotubes@NiCo_2_S_4_ nanosheets core-shell composites with improved electrochemical performance as supercapacitors; published by Elsevier, 2017 [144].

Dai et al. prepared relatively stable ZnCo_2_O_4_@Ni [171]. The specific capacity of Ni-Co-S composite electrode material was 1396.9 C g^−1^ at a current density of 1 A g^−1^, while ZnCo_2_O_4_ nanorods and Ni-Co-S showed a specific capacity of 1025.5 C g^−1^ and 1026 C g^−1^, respectively, under the same conditions. At the same time, the device showed a capacity retention rate of 85.5% after 1000 charge-discharge cycles at 4 A g^−1^. Bai et al. prepared 2D Co_3_O_4_@Ni(OH)_2_ [175]. The SC synthesized by this method had a specific capacitance of 98.4 F g^−1^ in the potential range of 0–1.7 V at 5 mA cm^−2^ and an energy density of 40.0 W h kg^−1^ at a power density of 349.6 W g^−1^. In addition, the original specific volume retention rate was 90.5% after 5000 cycles (1.61 A g^−1^). This proved that the composite with core-shell structure can retain the advantages of each component, and the synergistic effect between them can be used to improve the electrochemical properties of the material. Based on the above, Zhang et al. synthesized layered core-shell polyporrole nanotubes@NiCo_2_S_4_ materials by coating NiCo_2_S_4_ nanosheets on conductive polypyrrole nanotubes [144]; the formation process is shown in Figure 15. The material had a specific capacitance of 911 F g^−1^ at a current density of 1 A g^−1^ and maintained a capacitance of 592 F g^−1^ at a current density of 20 A g^−1^. After 4000 cycles at a current density of 5 A g^−1^, the original capacitance was 93.2%.

### 2.5. Other Cobalt-Containing Materials

Among other cobalt-containing materials, Co_3_O_4_@NiMoO_4_ has been most studied because NiMoO_4_ has good conductivity, which can improve the energy storage capacity of Co_3_O_4_.

Zhang et al. used hydrothermal and annealing methods to synthesize flower-like hybridized arrays on nickel foam [176]. Using Co_3_O_4_ nanowire arrays as scaffolds, NiMoO_4_ nanosheets were grown on the surface to form a new type of 3D layered battery electrode Co_3_O_4_@NiMoO_4_. The specific capacity of the hybrid array of the prepared Co_3_O_4_@NiMoO_4_ was 636.8 C g^−1^ at 5 mA cm^−2^. Moreover, the retention rate was 84.1% at 20 mA cm^−2^ after 2000 cycles and showed excellent electrochemical performance. The prepared hybrid capacitor (Co_3_O_4_@NiMoO_4_ as the positive electrode and activated carbon as the negative electrode) reached a high energy density of 58.5 W h kg^−1^ at 389 W kg^−1^.

Yang et al. adopted a similar method using mesoporous Co_3_O_4_ nanowires directly grown on the nickel foam as the skeleton to support the NiMoO_4_ nanosheet coating, and obtained Co_3_O_4_@NiMoO_4_ [177]. The high specific capacitance of the synthesized Co_3_O_4_@NiMoO_4_ was 3.61 F cm^−2^ at a current density of 2 mA cm^−2^. After 9000 cycles, about 101.3% of the initial capacity was still retained. Such a unique structure can significantly improve the permeability of electrolyte ions in the material.

Li et al. designed and synthesized nanowire/nanosheet arrays directly grown on carbon cloth by a two-step hydrothermal method [178]. Growing uniformly on carbon cloth collectors, the crystalline Co_3_O_4_ nanowires were used as backbone supports and provided reliable electrical connections for NiMoO_4_ nanosheet coatings with mesoporous structures. This enabled NiMoO_4_ to be fully utilized by creating faster electron/ion conductivity and electroactive sites. When the current density was 3 mA cm^−2^, the specific capacitance of the prepared 3D hybrid nanocomposites was 3.61 F cm^−2^, and when the current density increased from 3 mA cm^−2^ to 15 mA cm^−2^, the capacitance retention was 82%. The combined effect of the 3D nanostructure and the pseudo capacitance of the electrode materials resulted in superior electrochemical performance.

**Figure 16:**
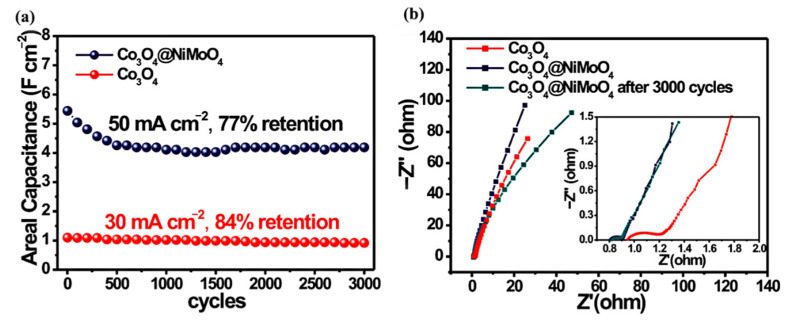
(**a**) Long-term cycling stability of the Co_3_O_4_ and Co_3_O_4_@NiMoO_4_ hybrid electrodes. (**b**) Impedance Nyquist plots of the Co_3_O_4_ electrode and the Co_3_O_4_@NiMoO_4_ hybrid electrode before and after 3000 cycles. Reproduced with permission from Daoping Cai, Three-Dimensional Co_3_O_4_@NiMoO_4_ Core/Shell Nanowire Arrays on Ni Foam for Electrochemical Energy Storage; published by ACS Publications, 2014 [179].

Cai et al. fabricated a 3D structure Co_3_O_4_@NiMoO_4_ using a similar method as above [179]. A shown in Figure 16, the prepared material showed a significantly enhanced surface capacitance of 5.69 F cm^−2^ when the current density was 30 mA cm^−2^, which was five times that of the original Co_3_O_4_ electrode (1.10 F cm^−2^). With a power density of 5000 W kg^−1^, the energy density of the hybrid electrode was 56.9 W h kg^−1^.

**Figure 17:**
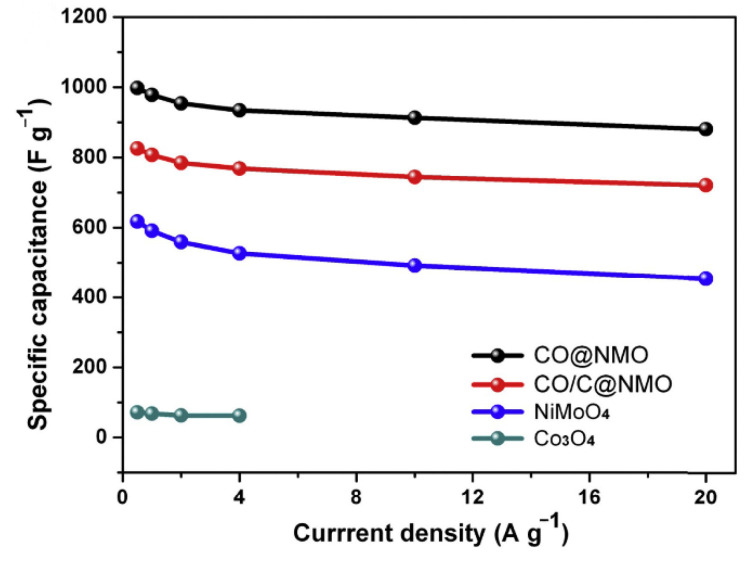
Specific capacitance of CO@NMO, CO/C@NMO, NiMoO_4_ andCo_3_O_4_ electrodes. Reproduced with permission from Ping Yang, Synthesis of hierarchical tube-like yolk-shell Co_3_O_4_@NiMoO_4_ for enhanced supercapacitor performance; published by Elsevier, 2018 [180].

Dong et al. first prepared a layered tubular yolk-shell composite by electrospinning and hydrothermal methods, and then calcination to prepare a Co_3_O_4_@NiMoO_4_ composite [180]. As shown in Figure 17, the Co_3_O_4_@NiMoO_4_ composite was made into an electrode with a specific enhanced capacitance of 913.25 F g^−1^ at a high current density of 10 A g^−1^, and a capacitance retention of 88% due to its unique structure and chemical composition. When the current density changes from 0.5 A g^−1^ to 20 A g^−1^, it had remarkable cycle stability.

Hong et al. prepared a uniform 2D Co_3_O_4_ structure by a simple chemical etching assisted method followed by thermal annealing, and then synthesized Co_3_O_4_@NiMoO_4_ by a simple hydrothermal method [181]. The specific capacitance of the 3D hybrid nanostructures was1526 F g^−1^ at the current density of 3 mA cm^−2^, and the capacitance retention was 72% when the current density increased from 3 mA cm^−2^ to 30 mA cm^−2^. On this basis, a Co_3_O_4_@NiMoO_4_ ASC was designed, and the maximum energy density of activated carbon was 37.8 W h kg^−1^ when the power density was 482 W kg^−1^.

The above describes another cobalt-containing material, Co_3_O_4_@NiMoO_4_. Among them, NiMoO_4_ can improve the electrochemical performance of Co_3_O_4_. The electrochemical performance and stability of the two materials can be greatly improved by rational design of their microstructure, which has great potential.

## 3. Summary and Outlook

In conclusion, this paper reviews the application of cobalt-based nanomaterials in supercapacitors and presents the contributions of many scholars in this field in recent years. These scholars have tried many approaches to improve the electrode materials and enhance the supercapacitor performance. The properties of cobalt-based materials and the issues related to supercapacitors are also discussed.

In this paper, we first introduce the classification and working principle of SCs. According to the charge storage mechanism of SCs, they can be classified into three categories: EDLCs, PCs and battery-type capacitors. EDLCs store charge through a physical adsorption process controlled by reversible adsorption/desorption of electrolyte ions at the electrode/electrolyte interface without any chemical reaction involved. In contrast, PCs and battery-type capacitors benefit from Faraday redox reactions and have a unique charge storage mechanism with much larger capacitance and power density than EDLCs. Among the many electrode materials for these SCs, common cobalt-based materials include cobalt oxide, cobalt hydroxide, and cobalt-containing ternary metal oxides. Among them, the theoretical specific capacitance of Co_3_O_4_ (3560 F g^−1^) is slightly higher than that of Co(OH)_2_ (3460 F g^−1^), and electrodes made from Co_3_O_4_ usually exhibit better cycling performance than that of Co(OH)_2_. Compared to these two substances, the ternary metal oxides (MnCo_2_O_4_, NiCo_2_O_4_ and ZnCo_2_O_4_) show significantly higher performance due to the synergistic effect of the two transition metals coupled together.

To further enhance the performance of the above cobalt-based materials, the main methods are: (1) designing the morphology of the electrode materials; (2) introducing other elements, such as S, P, and Mn, among others; (3) compounding with other materials, and (4) improving the preparation process. First, designing unique morphologies is an effective and commonly used means to enhance the electrochemical performance of electrode materials. Microstructures such as nanoparticles, nanowires, nanotubes, nanosheets, and nanospheres are mainly used in the many studies reported in this paper. Among these morphologies, mesoporous structures play a major role. On the one hand, a mesoporous structure can significantly increase the surface area and shorten the diffusion length for electron and ion transport, thus accelerating the redox process and improving pseudo-capacitance performance. On the other hand, it can moderate the volume change during the charging/discharging process, thus improving the cycling capability. Second, the introduction of other elements can further improve the performance of cobalt-based nanomaterials. As mentioned above, transition metal sulfides have significantly higher electrical conductivity and redox ability than corresponding metal oxides. Meanwhile, compounding cobalt-based nanomaterials with other materials can combine the advantages of both materials and improve the performance of electrodes. As mentioned above, many scholars have compounded cobalt-based nanomaterials with carbon-based materials, which are very commonly used today. Among the many carbon-based materials, graphene, which has a large specific surface area and excellent mechanical and electrochemical properties, is an ideal carrier. As a result, many graphene-cobalt-based nanomaterial composites have emerged in recent years. Finally, the electrode performance can also be enhanced by improving the current process. Among the many studies presented in this paper, hydrothermal methods have been widely used, which can easily alter the morphology and structure of nanomaterials. In addition, processes such as electrochemical deposition, electrostatic spinning, and sol-gel methods are also widely used due to their advantages in preparing nanostructures.

Some researchers have investigated the effects of some external factors (e.g., ultraviolet radiation, annealing temperature, deposition potential, etc.) on the performance of SCs. Ultraviolet irradiation increases the crystallinity of raw materials, and the electrochemical performance of supercapacitors made from ultraviolet-irradiated electrode materials was significantly improved [182]. During the annealing process, the grains agglomerate to form large particles, resulting in a uniform and dense porous microstructure [183]. This porous microstructure facilitates electrolytic ion insertion and electron transfer at the electrode/electrolyte interface, resulting in effective charge storage. As for the deposition potential, it has been shown that lower deposition potential leads to lower mass transfer rate and lower electrochemical performance.

In recent years, the field of energy storage devices has been developing rapidly, and sodium-ion batteries, potassium-ion batteries, and various kinds of SCs are being widely and deeply researched, among which miniature SCs (MSCs) are gradually attracting the attention of many researchers. MSCs are miniaturized SCs that have a similar composition to conventional SCs, but with significant structural differences. Conventional SCs have a vertical sandwich structure with inherent limitations including short-circuiting within a narrow distance between two electrodes, increased ion transport resistance, and high mass loading of active materials at an appropriately long distance [184,185]. By contrast, MSCs have a planar structure with a narrow insulating gap between the two electrodes, which avoids the use of a separator. This increases the mass loading of the active material, resulting in high power and energy density, low ion transport resistance, and short electrolyte ion diffusion distance [186]. Due to their small size and excellent electrochemical properties, MSCs could soon be widely used in various applications. Therefore, it is important to study the application of cobalt-based nanomaterials in MSCs.

It should also be noted that studies have shown that a deficit in cobalt supply could occur as early as 2030 [187]. This means that the advantage of the low cost of cobalt-based materials compared to RuO_2_ will gradually decrease. The solutions to this problem are as follows: (1) finding alternative materials, such as Ni, Mn, Zn, and other transition group metals with good performance; (2) hybridizing cobalt-based materials with conductive materials with good performance to reduce the content of cobalt in monoliths while ensuring performance (there have been studies on doping polyaniline, polypyrrole, carbon nanotubes, and graphene, among other substances, into cobalt-based materials), and (3) further developing more efficient, convenient, and low-cost SCs recycling technology.

## Figures and Tables

**Table 1:** Electrochemical properties of each microscopic morphology.

Morphology	Specific Capacitance (Current Density)	Cycling Performance (Cycles, Current Density)	Year	Ref.
nanofibers	407 F g^−1^ (5 mV s^−1^)	94% (1000, 1 A g^−1^)	2014	[51]
layered Co_3_O_4_	352 F g^−1^ (2 A g^−1^)	129% (2500, 2 A g^−1^)	2012	[52]
nanoparticles	362.8 F g^−1^ (0.2 A g^−1^)	73.5% (1000, 1 A g^−1^)	2014	[53]
nanorod arrays	154.9 C g^−1^ (1 A g^−1^)	88% (1000, 1 A g^−1^)	2019	[54]
core-shell Co_3_O_4_	837.7 F g^−1^ (1 A g^−1^)	87.0% (2000, 5 A g^−1^)	2018	[55]
porous nanowires	2815.7 F g^−1^ (1 A g^−1^)	88.8% (1100, 1 A g^−1^)	2018	[56]
hollow coral shape	626.5 F g^−1^ (5 mV s^−1^)	≈100% (5000, 10 A g^−1^)	2019	[57]

**Table 2:** Summary of materials, structures, and electrochemical properties of cobalt-containing ternary metal oxides.

Materials	Structure	Specific Capacitance (Current Density)	Cycling Performance (Cycles, Current Density)	Year	Refs.
MnCo_2_O_4_	polyhedral nanostructure	1763 F g^−1^ (1 A g^−1^)	95% (4500, 1 A g^−1^)	2017	[89]
	flower-like hollow microspheres	235.7 F g^−1^ (1 A g^−1^)	93.6% (2000, 1 A g^−1^)	2016	[87]
	3D porous structure	503 F g^−1^ (1 A g^−1^)	97.4% (5000, 10 A g^−1^)	2019	[93]
	belt-based core-shell nanoflowers	2154 F g^−1^ (5 A g^−1^)	90% (2500, 6 A g^−1^)	2016	[88]
NiCo_2_O_4_	hollow nanospheres with layered structure	1229 F g^−1^ (1 A g^−1^)	86.3% (3000, 50 mV s^−1^)	2018	[104]
	hollow spheres	1036 F g^−1^ (1 A g^−1^)	78.6% (10,000, 5 A g^−1^)	2015	[105]
	hollow sub microspheres	678 F g^−1^ (1 A g^−1^)	87% (3500, 10 A g^−1^)	2013	[107]
	urchin-like hollow microspheres	942.2 F g^−1^ (0.5 A g^−1^)	90% (1000, 2.5 mA cm^−2^)	2017	[108]
	mesoporous hollow microspheres	987 F g^−1^ (1 A g^−1^)	≈100% (5000, 5 A g^−1^)	2015	[109]
	3D porous graphene/NiCo_2_O_4_ hybrid films	708.36 F g^−1^ (1 A g^−1^)	94.3% (6000, 10 A g^−1^)	2020	[120]
	flower-like hollow	728.4 F g^−1^ (1 A g^−1^)	95.9% (1000, 8 A g^−1^)	2014	[121]
	ultra-thin nanosheets	1801 F g^−1^ (1 mA cm^−2^)	90.9% (2000, 20 mA cm^−2^)	2016	[122]
	3D layered nuclear shell nanowires/nanowires sheet array	__	85.2% (3000, 20 mA cm^−2^)	2015	[123]
	layered nanostructure	1152 F g^−1^ (1 A g^−1^)	95.38% (3000, 6 A g^−1^)	2018	[124]
	layered core-shell nanostructures	2045.2 F g^−1^ (1 A g^−1^)	85.3% (21000, 4 A g^−1^)	2018	[94]
ZnCo_2_O_4_	nanowire	1625 F g^−1^ (5 A g^−1^)	94% (5000, 20 A g^−1^)	2014	[132]
	porous structure	776.2 F g^−1^ (1 A g^−1^)	84.3% (1500, 3 A g^−1^)	2017	[133]
	hexagonal like nano materials	845.7 F g^−1^ (1 A g^−1^)	95.3% (5000, 5 A g^−1^)	2017	[134]
	3D layered peony flower like material	440 F g^−1^ (1 A g^−1^)	155.6% (3000, 2 A g^−1^)	2017	[135]
	2D nanosheets	2111 F g^−1^ (1 A g^−1^)	93% (3000, 2 A g^−1^)	2021	[136]
	nanowires	48.6 C g^−1^ (1 A g^−1^)	90.5% (10,000, 1 A g^−1^)	2021	[137]
	nanowire array with core-shell structure	13.4 F cm^−2^ (4 mA cm^−2^)	98.5% (5000, 100 mA cm^−2^)	2018	[138]

## Data Availability

Not applicable.
